# Motor Deficits After Insular Glioma Surgery: Association with Postoperative Ischemia and Intraoperative Neuromonitoring

**DOI:** 10.3390/cancers18213583

**Published:** 2026-11-07

**Authors:** Gerard Plans, Isabel Fernández-Conejero, Daniel Martí, Pere J. Cifre-Serra, Albert Pons-Escoda, Alejandro Fernández-Coello, Júlia Miró, Aleix Rosselló, Andreu Gabarrós

**Affiliations:** 1 Department of Neurosurgery, Hospital Universitari de Bellvitge, L’Hospitalet de Llobregat, 08907 Barcelona, Spain; dmartid@bellvitgehospital.cat (D.M.); pcifre@bellvitgehospital.cat (P.J.C.-S.); afernandezc@bellvitgehospital.cat (A.F.-C.); a.rossello@bellvitgehospital.cat (A.R.); agabarros@bellvitgehospital.cat (A.G.); 2 Facultat de Medicina i Ciències de la Salut, Universitat de Barcelona (UB), 08036 Barcelona, Spain; isabelfernandez@bellvitgehospital.cat (I.F.-C.); albert.pons@bellvitgehospital.cat (A.P.-E.); jmiro@bellvitgehospital.cat (J.M.); 3 Institut d’Investigació Biomèdica de Bellvitge (IDIBELL), L’Hospitalet de Llobregat, 08908 Barcelona, Spain; 4 Neuro-Oncology Unit, Hospital Universitari de Bellvitge, L’Hospitalet de Llobregat, 08907 Barcelona, Spain; 5 Department of Neurology, Hospital Universitari de Bellvitge, L’Hospitalet de Llobregat, 08907 Barcelona, Spain; 6 Department of Radiology, Hospital Universitari de Bellvitge, L’Hospitalet de Llobregat, 08907 Barcelona, Spain; 7 Institut de Diagnòstic per la Imatge (IDI), L’Hospitalet de Llobregat, 08908 Barcelona, Spain

**Keywords:** insular glioma, motor deficits, postoperative ischemia, intraoperative neuromonitoring

## Abstract

Background and Objectives: The proximity of insular gliomas to eloquent brain structures and complex vascular anatomy makes preservation of neurological function during resection challenging. This study investigates postoperative motor deficits after insular glioma resection and their association with intraoperative neuromonitoring and ischemia. Methods: In total, 64 patients who underwent insular glioma resection between 2018 and 2025 were included. Extent of resection (EOR), intraoperative MEP changes, subcortical stimulation thresholds, postoperative ischemic lesions and motor outcomes at 3 months were evaluated. Results: Median EOR was 93.4% (IQR 87.4–100%), and median residual tumor volume was 4.5 cm^3^ (IQR 0–8.1). MEP monitoring was performed in 56 patients (87.5%); deterioration occurred in 3 (5.4%), all of whom developed persistent deficits. MEP deterioration showed 60% sensitivity (95% CI 14.7–94.7%), 100% specificity (95% CI 93.0–100%), 100% positive predictive value (95% CI 29.2–100%), and 96.2% negative predictive value (95% CI 87.0–99.5%). The positive predictive value should be interpreted cautiously given the small number of events. Subcortical responses were elicited in 48 patients (85.7%). Tumors involving Berger–Sanai zones II and/or III had lower subcortical stimulation thresholds than tumors sparing both regions (median 4 vs. 8.5 mA; *p* = 0.012), although thresholds were not associated with motor deficits or ischemia. Postoperative ischemic lesions occurred in 20 patients (31.3%). Persistent motor deficits occurred in 6 patients (9.4%), all with associated ischemic lesions. Conclusions: Postoperative motor deficits were associated with ischemic lesions. MEP deterioration showed high specificity and positive predictive value, but limited sensitivity. Subcortical stimulation provided functional information about corticospinal tract proximity. These findings highlight the importance of preserving vascular supply to motor pathways during insular glioma surgery.

## Simple Summary

Insular glioma surgery carries a risk of motor deficits due to the proximity of the corticospinal tract and deep perforating arteries. This study investigates the relationship between intraoperative motor monitoring, postoperative ischemia, and persistent motor deficits. The findings suggest that the described intraoperative monitoring and stimulation technique helps identify the anatomical location of the corticospinal tract and supports its functional preservation during resection. However, all patients with persistent postoperative motor deficits had ischemic lesions, highlighting the importance of preserving perforating arteries arising from the middle cerebral artery. Intraoperative motor monitoring remains essential for assessing motor pathway function, but stable signals do not completely exclude postoperative injury, as false-negative results occurred. These findings emphasize that functional monitoring and meticulous vascular preservation must be complementary components of surgical strategy. Further refinement of monitoring techniques is needed to improve sensitivity and reduce undetected injuries, potentially enhancing neurological outcomes after insular glioma surgery.

## 1. Introduction

Insular gliomas represent one of the most challenging tumors in neurosurgery due to their deep location and intimate relationship with critical neurovascular and functional structures. The insula is surrounded by the frontal, parietal, and temporal opercula and is closely associated with white matter pathways involved in language and motor function. Moreover, it is traversed by branches of the MCA and its perforating vessels, increasing the risk of neurological deficits and vascular complications during tumor resection.

The primary goal of surgical treatment is maximal safe resection while preserving neurological function. Increasing evidence indicates that EOR is a key prognostic factor in glioma surgery and has been consistently associated with improved survival [1,2,3,4,5] and seizure control [6,7,8]. However, the infiltrative nature of these tumors and their proximity to eloquent cortical and subcortical pathways often limit the extent of resection.

Advances in microsurgical techniques and intraoperative technologies, including neuronavigation, functional imaging, brain mapping, and intraoperative neurophysiological monitoring (IONM), have improved surgical safety. Nevertheless, postoperative neurological deficits remain a significant concern, with motor deficits reported in 2–20% of cases [5,8,9,10,11].

Although ischemic injury is a well-recognized cause of postoperative motor deficits, the relative contribution of vascular versus direct corticospinal tract damage remains insufficiently characterized.

The aim of this study was to analyze our institutional experience in insular glioma surgery, focusing on extent of resection, the occurrence and mechanisms of postoperative motor deficits, and their relationship with IONM findings and postoperative ischemia.

## 2. Materials and Methods

### 2.1. Study Design and Patient Population

We conducted a retrospective study of patients who underwent surgical resection of insular gliomas at our institution between 2018 and 2025. Clinical, radiological, surgical and IONM data were obtained from a prospectively maintained database.

Patients were identified through a systematic search of the institutional database. Inclusion criteria were histopathologically confirmed insular glioma, availability of preoperative and postoperative MRI, intraoperative functional assessment through neurophysiological monitoring, awake cortical and subcortical mapping, or a combination of both techniques, and complete clinical follow-up at 3 months after surgery.

Demographic, clinical, tumor-related, surgical, IONM, and postoperative neurological outcome data were systematically reviewed, with particular focus on motor deficits and their potential underlying mechanisms.

The study was approved by the Clinical Research Ethics Committee of Bellvitge University Hospital, which considered that informed consent was not required given the retrospective nature of the study.

### 2.2. Radiological Assessment

All patients underwent preoperative magnetic resonance imaging (MRI) using 1.5- or 3-T scanners (Philips Healthcare, Best, The Netherlands), including T1-weighted imaging (WI), T2-WI, T2-FLAIR, contrast-enhanced (CE) T1-WI, and diffusion-weighted imaging (DWI) with standard b values of 0 and 1000 s/mm^2^, with automatic generation of apparent diffusion coefficient (ADC) maps. Tumor location, radiological characteristics, and relationships with surrounding structures were assessed. Insular involvement was classified according to the Berger–Sanai classification [2].

Diffusion tensor imaging (DTI) was acquired using a single-shot echo-planar imaging sequence with diffusion gradients applied in 32–64 directions (b = 800–1000 s/mm^2^). DTI tractography was performed using dedicated planning software (Brainlab Elements, Brainlab AG, Munich, Germany) to reconstruct major white matter pathways, including language-related tracts and the corticospinal tract, for surgical planning.

Tumor volumes were calculated using the same software based on manual segmentation, and EOR was determined by comparing preoperative and postoperative imaging.

Early postoperative MRI was performed within 72 h after surgery to assess the extent of resection and identify postoperative ischemic lesions. Gross total resection was defined as the absence of radiologically detectable residual tumor on postoperative MRI, whereas subtotal resection was defined as the presence of any measurable residual tumor. Ischemic lesions were defined as areas of restricted diffusion on DWI with corresponding low signal on ADC maps. Ischemic lesions were reviewed and classified according to their anatomical distribution and presumed vascular territory.

### 2.3. Surgical Technique

The surgical strategy was tailored according to tumor location and its relationship with eloquent brain areas. Patients with tumors located in the non-dominant hemisphere were operated on under general anesthesia with IONM.

Tumors involving the dominant hemisphere were generally treated using an asleep–awake–asleep technique with cortical and subcortical language mapping. The final stage of the surgery was performed again under general anesthesia, allowing continuous IONM to control motor function during tumor resection. In selected cases surgery was performed fully awake depending on patient tolerance.

The surgical approach was determined based on tumor location, involvement of the opercular cortex and intraoperative findings during functional mapping, using either a transopercular or transsylvian route.

Tumor resection was carried out using standard microsurgical techniques, including bipolar coagulation, suction, and ultrasonic aspiration. Neuronavigation (Brainlab, Brainlab AG, Munich, Germany) was used in all cases. Topical papaverine was selectively applied when intraoperative findings suggested possible vascular compromise, particularly when vessel manipulation appeared to result in a reduction in vessel diameter consistent with vasospasm. It was also considered in cases of MEP deterioration or loss when an identifiable vessel showed signs of vasospasm, with the aim of achieving vasodilation and potentially improving cerebral perfusion.

The aim of surgery was to achieve maximal safe resection while preserving neurological function. 

### 2.4. Intraoperative Mapping and Monitoring

#### 2.4.1. Intraoperative Neuromonitoring Under General Anesthesia

Patients operated under general anesthesia received total intravenous anesthesia with propofol and remifentanil in order to optimize IONM. Muscle relaxants were used only for orotracheal intubation.

Multimodal IONM was performed using a 32-channel ISIS system (Inomed Co., Emmendingen, Germany). Motor-evoked potentials (MEPs) were obtained using transcranial electrical stimulation (TES) and direct cortical stimulation (DCS). TES was also used to assess the functional integrity of the contralateral hemisphere and to rule out anesthetic-related causes in case of intraoperative loss of MEP responses.

A high-frequency monopolar anodal stimulation using a train of five pulses (0.5 ms pulse duration and 4 ms interstimulus interval) was applied for both TES and DCS.

MEPs elicited by TES were obtained through stimulation via corkscrew electrodes placed on the scalp over the motor cortex according to the International 10–20 EEG system. MEPs elicited by DCS were obtained using an 8-contact strip electrode placed over the motor cortex with the aid of neuronavigation.

Responses were recorded from distal muscles of the upper and lower extremities. Stimulation intensity was adjusted to the threshold level, defined as the minimum intensity required to obtain reliable MEPs, and was generally kept below 25 mA to minimize the risk of stimulation-induced seizures.

Continuous monitoring of MEPs during tumor resection was combined with cathodal subcortical stimulation using a monopolar probe to estimate the distance to the corticospinal tract.

MEP stimulation and recordings were maintained continuously throughout tumor resection and continued until the beginning of dural closure.

Significant intraoperative changes were defined as an increase in stimulation threshold ≥ 5 mA, a reduction in MEP amplitude greater than 50%, or complete loss of responses. When these occurred, corrective measures were undertaken, including temporary interruption of resection, optimization of systemic blood pressure, irrigation with warm saline, and topical papaverine in cases of suspected vascular compromise. Tumor resection was resumed if MEPs recovered and discontinued if changes persisted.

Subcortical stimulation was initially performed using high intensities (up to 20 mA) and was considered negative if no response was obtained. Subcortical stimulation thresholds were used as a surgical guide, with low thresholds (≤5 mA) considered a warning sign. Resection was continued only if complete tumor removal was considered feasible based on preoperative imaging, intraoperative findings, or neuronavigation guidance.

For each patient, the minimum scMEP stimulation threshold recorded at any point during tumor resection was used for analysis. When multiple thresholds were obtained at different locations along the resection cavity, the lowest value was retained. Although this minimum threshold was generally obtained near the final resection margin, it could correspond to another location within the resection cavity.

The IONM protocol used in this study has been previously described in detail by our group and is summarized here for completeness [12].

#### 2.4.2. Awake Mapping Technique

In patients undergoing awake surgery, cortical and subcortical mapping was performed to identify and preserve eloquent motor and language areas during tumor resection. After craniotomy and dural opening, patients were awakened and functional mapping was initiated.

Electrical stimulation mapping (ESM) was performed using a bipolar probe (5 mm interelectrode distance) delivering biphasic current (60 Hz, 1 ms pulse duration, 3 s trains) with an Ojemann cortical stimulator (Radionics, Inc., Burlington, MA, USA) or an ISIS system (Inomed Co., Emmendingen, Germany). Stimulation intensity was progressively increased in steps of 0.5 mA starting at 1 mA, and the minimum effective current required to elicit a motor or language response was subsequently used throughout the procedure.

Cortical mapping was used to identify motor and language areas. Motor sites were defined by stimulation-induced muscle contraction or speech arrest, whereas language sites were identified by speech disturbances during standardized tasks, including object naming and speech production under neuropsychological supervision. Positive sites were marked and preserved.

During tumor resection, language function was continuously assessed with naming tasks combined with phonologic or semantic tasks. Subcortical stimulation was performed using the same parameters, and tumor resection was halted when language disturbances occurred to avoid permanent deficits.

In selected cases, surgery was performed fully awake, whereas in most patients the final stage of resection was completed under general anesthesia with IONM.

### 2.5. Histopathological Evaluation

Histopathological diagnosis was established according to the 2021 World Health Organization (WHO) classification of tumors of the central nervous system [13]. All tumor specimens were reviewed by experienced neuropathologists.

Tumors diagnosed before the publication of the 2021 WHO classification were retrospectively reclassified according to the updated diagnostic criteria when sufficient histopathological and molecular information was available.

Tumor grading and classification were therefore reported in accordance with the 2021 WHO criteria.

### 2.6. Clinical Outcome Assessment

Postoperative motor function was assessed during the first 24 h after surgery and at 3 months. Immediate postoperative motor deficits were defined as new motor impairments documented within the first 24 h after surgery, whereas persistent motor deficits were defined as motor impairments still present at the 3-month assessment. Motor deficit was defined as any new or worsened postoperative motor weakness on clinical examination and was graded according to the Medical Research Council (MRC) scale. Severity was further categorized using the Karnofsky Performance Status (KPS) scale. Postoperative deficits were classified as severe when the KPS score was below 70 and as mild or moderate when the KPS score was ≥70. The Karnofsky Performance Status (KPS) was used to assess the overall functional impact of postoperative neurological impairment, rather than motor deficit severity in isolation. This approach has been previously used in our work on glioma surgery [12]. Postoperative MRI scans were reviewed to identify potential causes of motor deficits, including ischemic lesions. Intraoperative neuromonitoring (IONM) findings were compared with functional status at 3 months to identify intraoperative neurophysiological predictors of postoperative motor deterioration.

### 2.7. Statistical Analysis

Descriptive statistics were used to summarize demographic, clinical, radiological, surgical, and intraoperative monitoring variables. Continuous variables are presented as mean ± standard deviation or median with interquartile range (IQR) when appropriate, while categorical variables are reported as frequencies and percentages.

Comparisons between groups were performed to evaluate factors associated with postoperative motor deficits. Continuous variables were assessed for normality using the Shapiro–Wilk test. Normally distributed continuous variables were compared using Welch’s t-test, whereas non-normally distributed continuous variables were compared using the Mann–Whitney U test. Categorical variables were compared using Pearson’s chi-square test with continuity correction when appropriate, or Fisher’s exact test when expected cell frequencies were small. The diagnostic performance of intraoperative MEP deterioration for predicting postoperative motor deficits was assessed by calculating sensitivity, specificity, positive predictive value, and negative predictive value.

Statistical significance was defined as a *p* value < 0.05. Statistical analyses were performed using IBM SPSS Statistics for Windows, Version 26.0 (IBM Corp., Armonk, NY, USA).

## 3. Results

### 3.1. Patient Characteristics

A total of 64 patients with insular gliomas who underwent surgical resection at our institution were included in the study. The median age at surgery was 47 years (IQR 38–57.8 years) and 38 patients (59%) were male.

Tumors were located in the left hemisphere in 36 patients (56.2%) and in the right hemisphere in 28 patients (43.8%). The most common presenting symptom was seizures (65%), followed by neurological deficits (21%) and headache or signs of intracranial hypertension (14%). Less frequent presentations included dizziness or imbalance (2%), and 2% of tumors were incidentally diagnosed.

### 3.2. Tumor Characteristics

The median preoperative tumor volume was 58.2 cm^3^ (IQR 43.5–82.3 cm^3^).

According to the 2021 WHO classification [13], glioblastoma was the most frequent histological diagnosis (28 patients, 43.75%), followed by astrocytoma grade 3 (13 patients, 20.3%) and astrocytoma grade 2 (11 patients, 17.2%). Oligodendroglioma grade 3 was identified in 8 patients (12.5%), while oligodendroglioma grade 2 accounted for 2 cases (3.1%). Two patients had astrocytoma grade 4 (3%).

Overall, high-grade gliomas accounted for the majority of tumors in the series (51 patients, 79.7%), whereas low-grade gliomas represented 13 cases (20.3%).

Baseline patient and tumor characteristics are summarized in Table 1.

According to the Berger–Sanai classification, tumors involved the entire insula in 36 patients (56.3%). Among partially insular tumors, the most frequent pattern was combined involvement of zones I and IV (10 patients, 15.6%), followed by zones III and IV (5 patients, 7.8%) and zones II and III (3 patients, 4.7%). Isolated involvement of a single insular zone was uncommon. Overall, most tumors involved multiple insular zones.

### 3.3. Surgical Results

Tumor resection was performed through a transopercular approach in 44 patients (68.8%), including 20 temporal, 14 frontotemporal, and 10 frontal opercular approaches, whereas a transsylvian approach was used in 20 patients (31.2%).

The surgical approach differed according to tumor laterality. In right-sided tumors, the transopercular approach was used in 24 cases (85.7%) and the transsylvian approach in 4 cases (14.3%). In contrast, left-sided tumors were more frequently treated using the transsylvian approach (16 cases, 44.4%). This association between tumor hemisphere and surgical approach was statistically significant (chi-square = 5.34, *p* = 0.021).

The median EOR was 93.4% (IQR 87.4–100%), ranging from 22% to 100%. A resection ≥ 90% was achieved in 39 patients (60.9%), including 22 cases (34.4%) with complete tumor removal. Surgical characteristics and EOR are summarized in Table 2.

EOR did not significantly differ according to surgical approach or tumor laterality. The median EOR was 93.6% in patients treated through a transopercular approach and 92.8% in those undergoing a transsylvian approach, with no significant difference (Mann–Whitney U test, *p* = 0.584). Similarly, median EOR was 96% in right-sided tumors and 90.6% in left-sided tumors with no significant difference (Mann–Whitney U test, *p* = 0.145). Tumor grade was not associated with EOR, median EOR was 91.9% in high-grade and 94% in low-grade tumors, with no significant difference (Mann–Whitney U test, *p* = 0.753).

The median postoperative residual tumor volume was 4.5 cm^3^ (IQR 0–8.1 cm^3^), ranging from 0 to 77.5 cm^3^. A residual tumor volume < 5 cm^3^ was observed in 33 patients (51.6%), and <10 cm^3^ in 51 patients (79.7%). Residual tumor volume did not differ significantly between tumor grades with a median of 4.73 cm^3^ in high-grade and 1.42 cm^3^ in low-grade tumors (Mann–Whitney U test, *p* = 0.759).

IONM was used in 56 patients (87.5%), while fully awake surgery was performed in 8 cases (12.5%). MEPs remained stable in 53 cases (94.6%), whereas intraoperative MEP loss or significant MEP deterioration occurred in 3 patients (5.4%).

Subcortical motor mapping using high-frequency monopolar stimulation was performed in 56 patients. A positive subcortical motor response was obtained in 48 cases (85.7%), enabling identification of the corticospinal tract, whereas stimulation remained negative in eight patients (14.3%). A stimulation threshold ≤ 5 mA was observed in 25 patients (44.6%) (Table 3).

Among patients with an elicitable and quantifiable subcortical motor response (*n* = 48), tumors involving Berger–Sanai zones II and/or III, including those with total insular involvement, showed significantly lower stimulation thresholds than tumors sparing both regions: median 4 mA (IQR 3–7 mA) vs. 8.5 mA (IQR 5.75–10.75 mA) respectively (Mann–Whitney U test, *p* = 0.012). Cases with negative subcortical stimulation responses and cases in which subcortical stimulation was not performed were excluded from this analysis.

Immediate postoperative motor deficits documented within the first 24 h after surgery were identified in 12 patients (18.8%). Surgery-related persistent postoperative motor deficits at 3 months were observed in 6 patients (9.4%). Among patients with stable intraoperative MEPs, 2 (3.8%) developed a persistent motor deficit, whereas all patients with intraoperative MEP deterioration developed postoperative motor deficits (100%). An additional patient who underwent fully awake surgery without intraoperative neuromonitoring developed a persistent postoperative motor deficit. All these findings are summarized in Table 4.

Among the 56 patients undergoing MEP monitoring, the association between intraoperative MEP deterioration and postoperative motor deficits was statistically significant (Fisher’s exact test, *p* < 0.001).

In this subgroup, intraoperative MEP deterioration showed a positive predictive value of 100% (95% CI 29.2–100%) and a negative predictive value of 96.2% (95% CI 87.0–99.5%) for postoperative motor deficits. Sensitivity was 60% (95% CI 14.7–94.7%) and specificity was 100% (95% CI 93.0–100%). Diagnostic performance measures (sensitivity, specificity, positive predictive value, and negative predictive value) were calculated exclusively in the 56 patients who underwent intraoperative MEP monitoring. The eight patients who underwent fully awake surgery without continuous MEP monitoring were excluded from these analyses.

Regarding postoperative motor outcomes, persistent motor deficits at 3 months occurred in 5 of 49 patients (10.2%) with tumors involving Berger–Sanai zones II and/or III, including those with total insular involvement, and in 1 of 15 patients (6.7%) whose tumors spared both regions, with no statistically significant difference between groups (Fisher’s exact test, *p* = 1.000). Preoperative tumor volume did not differ significantly between patients with and without persistent motor deficits at 3 months: median 56.7 cm^3^ (IQR 54.8–82.7) vs. 59.6 cm^3^ (IQR 42.1–80.1), respectively (Mann–Whitney U test, *p* = 0.678).

Postoperative ischemic lesions were observed in 20 patients (31.3%). Most lesions involved the long perforating arteries of the middle cerebral artery (LPA-MCA) (17 cases, 85%), including 15 isolated LPA-MCA infarctions and 2 cases with concomitant infarction in the territory of anterior cerebral artery (ACA) perforating vessels. Isolated ischemia in the ACA territory was observed in 1 patient (5%). Infarcts related to the lateral lenticulostriate arteries (LLA) were identified in 2 patients (10%).

The incidence of postoperative ischemia was not associated with tumor grade or surgical approach. Ischemia occurred in 29.4% of high-grade and 38.5% of low-grade tumors (Fisher’s exact test, *p* = 0.523), and in 34.1% of transopercular versus 25.0% of transsylvian approaches (Pearson’s chi-square test with continuity correction, *p* = 0.663).

No significant association was found between subcortical stimulation threshold and either persistent postoperative motor deficit or postoperative ischemia when analyzed as a continuous variable (Mann–Whitney U test, *p* = 0.785 and *p* = 0.596, respectively). Similarly, using a threshold of ≤5 mA, no significant association was observed with either motor deficit (3/25 vs. 2/23, Fisher’s exact test, *p* = 1.000) or postoperative ischemia (9/25 vs. 7/23, Fisher’s exact test, *p* = 0.765).

**Figure 1:**
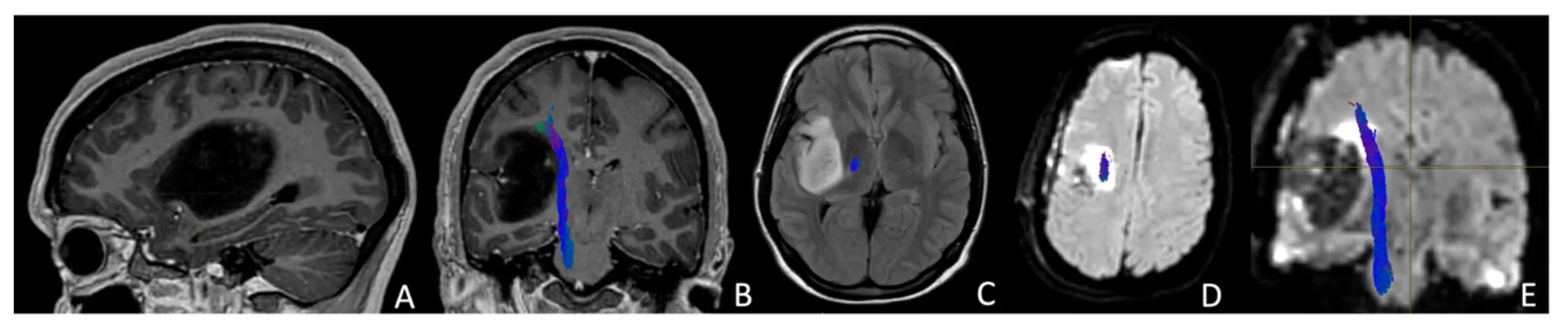
**Postoperative ischemic lesion associated with motor deficit.** A 38-year-old woman with a right insular glioma involving the entire insula underwent resection under general anesthesia via a frontotemporal transopercular approach. The subcortical MEP threshold was 3 mA. Cortical MEPs were lost during resection of the posterosuperior portion of the tumor. Postoperatively, the patient developed complete hemiplegia, which improved with rehabilitation. At 3-month follow-up, she was able to walk without assistance, with a mild residual motor deficit (KPS 80). (**A**) Preoperative sagittal T1-weighted MRI showing right insular glioma. (**B**) Preoperative coronal T1-weighted image with diffusion tensor imaging (DTI) tractography demonstrating the corticospinal tract (CST, blue), displaced and in close proximity to the tumor. (**C**) Preoperative axial FLAIR image showing the anatomical relationship between the CST and the tumor. (**D**) Immediate postoperative diffusion-weighted imaging (DWI) demonstrating an ischemic lesion in the deep white matter adjacent to the CST. (**E**) Coronal reconstruction of DWI showing the ischemic lesion at the superior margin of the resection cavity. **Abbreviations**: MEP, motor-evoked potential; DTI, diffusion tensor imaging; CST, corticospinal tract; DWI, diffusion-weighted imaging; KPS, Karnofsky Performance Status. **Notes:** In panels D and E, the CST representation corresponds to the preoperative DTI tractography, co-registered with the preoperative MRI and fused with the immediate postoperative MRI for anatomical reference. No postoperative DTI tractography was performed.

All postoperative motor deficits at 3 months were associated with ischemic lesions. This association was statistically significant (Fisher’s exact test, *p* < 0.001).Among patients with ischemia, 6 (30%) developed a persistent motor deficit at 3 months (Figure 1).

The occurrence and severity of motor deficits varied according to the vascular territory involved. Infarcts involving the LLA were associated with motor deficits (2/2 cases) and resulted in more severe functional impairment, with KPS < 70 in both patients. In contrast, ischemia involving the LPA-MCA resulted in motor deficits in 4 of 17 patients (23.5%), which were generally milder. These findings are summarized in Table 5.

The clinical, neurophysiological, and radiological characteristics of patients with persistent postoperative motor deficits are summarized in Table 6.

## 4. Discussion

Surgery plays a central role in the management of insular gliomas, with numerous studies demonstrating that a greater EOR is associated with improved overall survival (OS) [2,3,5,14] and progression-free survival (PFS) [2,5]. In both low- and high-grade insular gliomas, improved seizure control has also been associated with more extensive tumor removal [6,7,8,15]. In insular low-grade gliomas, maximal resection has additionally been linked to delayed malignant transformation [2,5].

In the present series, a high median EOR was achieved (93.4%; IQR 87.4–100%), with a median postoperative residual tumor volume of 4.5 cm^3^ (IQR 0–8.1 cm^3^). Complete tumor resection was obtained in 34.4% of patients. These resection rates are consistent with those reported in the literature [2,3,5,8,15,16,17,18,19] particularly considering that tumors in our series frequently involved multiple insular zones according to the Berger–Sanai classification. Despite the anatomical complexity of the insular region and its close relationship with critical vascular and subcortical structures, these results demonstrate that extensive tumor removal can be safely achieved in a substantial proportion of cases.

Overall, our findings support the concept of maximal safe resection as the primary surgical objective in insular glioma surgery and are consistent with previously reported series.

Although anatomical studies have suggested that the transopercular approach provides better exposure of the insula [14,20] no association was found in our series between extent of resection and surgical approach. This finding suggests that, with appropriate microsurgical technique and functional guidance, comparable resection rates can be achieved regardless of the chosen corridor.

In our experience, the transsylvian approach was significantly more frequent in tumors located in the dominant hemisphere, reflecting a surgical strategy aimed at preserving language function by minimizing manipulation of the opercular cortex.

Postoperative motor deficits were observed in 9.4% of patients in our series. This rate is consistent with previously published studies [3,5,8,11,15,16,19,21].

In our series, IONM findings were associated with postoperative motor outcomes, with MEP deterioration showing high specificity but limited sensitivity. The technique was first described and applied for the surgical resection of brain tumors under general anesthesia by Taniguchi in 1993 [22]. Since then, some papers have been published showing the usefulness of the technique for motor cortical mapping [12,23,24,25,26,27,28], motor subcortical mapping [12,25,29,30,31,32] and IONM with a low incidence of seizures [12,25,31,33,34,35]. Subcortical stimulation provides anatomical information regarding the proximity of the surgical cavity to the CST, with lower stimulation thresholds generally indicating closer proximity to the CST. However, this measurement does not directly assess functional injury to the CST. Conversely, continuous MEP monitoring provides dynamic functional information regarding corticospinal pathway integrity throughout tumor resection. These techniques therefore provide complementary information about the functional status of the CST during tumor resection.

In our series, motor-evoked potentials (MEPs) demonstrated a high predictive value, with a positive predictive value of 100% and a negative predictive value of 96.2%. However, sensitivity was limited to 60% with two deficits occurring despite stable MEPs.

Two patients developed motor deficits despite stable intraoperative MEPs, representing false-negative cases. These two false-negative cases deserve particular consideration. In the first patient, who had a tumor in the dominant hemisphere, we suspect that the vascular event may have occurred during the transition from the awake to the asleep phase of surgery, before MEP monitoring was initiated. The unusually high cortical stimulation intensity required to elicit MEPs (25 mA) from the very beginning of monitoring raises the possibility that functional impairment of the motor pathway was already present when monitoring was initiated. Moreover, the high stimulation intensity may have resulted in activation of motor pathway fibers beyond the ischemic region, potentially bypassing the affected area and explaining the absence of subsequent MEP deterioration. The subcortical stimulation threshold in this patient was 9 mA, and the ischemic lesion was located in the corona radiata.

In the second patient, no definite intraoperative event or technical explanation for the false-negative finding could be identified. The subcortical stimulation threshold was 12 mA, suggesting a relatively greater distance from the CST. The occurrence of a postoperative ischemic lesion and persistent motor deficit despite stable MEP responses is compatible with a vascular mechanism that may not have been detected by intraoperative monitoring.

False-negative findings in intraoperative neuromonitoring have been reported by other authors [19,35]. These findings underscore the need for further refinement of current monitoring techniques.

Subcortical motor mapping using high-frequency monopolar stimulation has been widely described as a reliable technique for identifying and preserving the CST during glioma surgery. Compared with bipolar 60 Hz stimulation, this approach has been shown to provide greater sensitivity in detecting the CST [29,32]. The stimulation threshold is distance-dependent, with an approximate relationship of 1 mA per 1 mm from the CST, and the risk of mechanical tract injury increases as the stimulation threshold decreases [12]. Although the safe subcortical threshold remains variable across studies, values in the range of 3–5 mA are generally considered indicative of close proximity to the CST [12,31,32].

In our cohort, a positive subcortical motor response was obtained in 85.7% of cases in which the technique was used, allowing tumor resection to be performed in close proximity to the CST. Low stimulation thresholds were frequently observed, reflecting the intimate anatomical relationship between insular gliomas and the corticospinal tract. Nevertheless, subcortical stimulation provided intraoperative guidance for identifying the CST and limiting the risk of direct tract injury.

Interestingly, although tumors involving Berger–Sanai zones II and/or III were associated with significantly lower subcortical stimulation thresholds, reflecting closer proximity to the corticospinal tract, this anatomical distribution was not associated with a higher incidence of persistent postoperative motor deficits. Similarly, preoperative tumor volume did not differ significantly between patients with and without persistent motor deficits. These findings suggest that anatomical proximity to the corticospinal tract alone may not adequately explain the occurrence of persistent motor deficits following insular glioma surgery. In the context of our finding that all patients with persistent motor deficits had postoperative ischemic lesions, these results support the potential contribution of vascular injury to postoperative motor deterioration, rather than direct mechanical injury to the corticospinal tract as the sole underlying mechanism. Nevertheless, a concomitant direct mechanical component cannot be excluded, and the retrospective design and limited number of motor deficit events preclude definitive conclusions regarding the relative contribution of these mechanisms.

In our series, all patients with persistent postoperative motor deficits had postoperative ischemic lesions, including those with low subcortical stimulation thresholds. These findings suggest that vascular injury, particularly involving the deep perforating arteries, may play an important role in the development of persistent motor deficits after insular glioma surgery. They also reinforce the value of contemporary intraoperative mapping techniques in preserving the corticospinal tract, while highlighting the importance of vascular preservation as an additional determinant of functional outcome.

**Figure 2:**
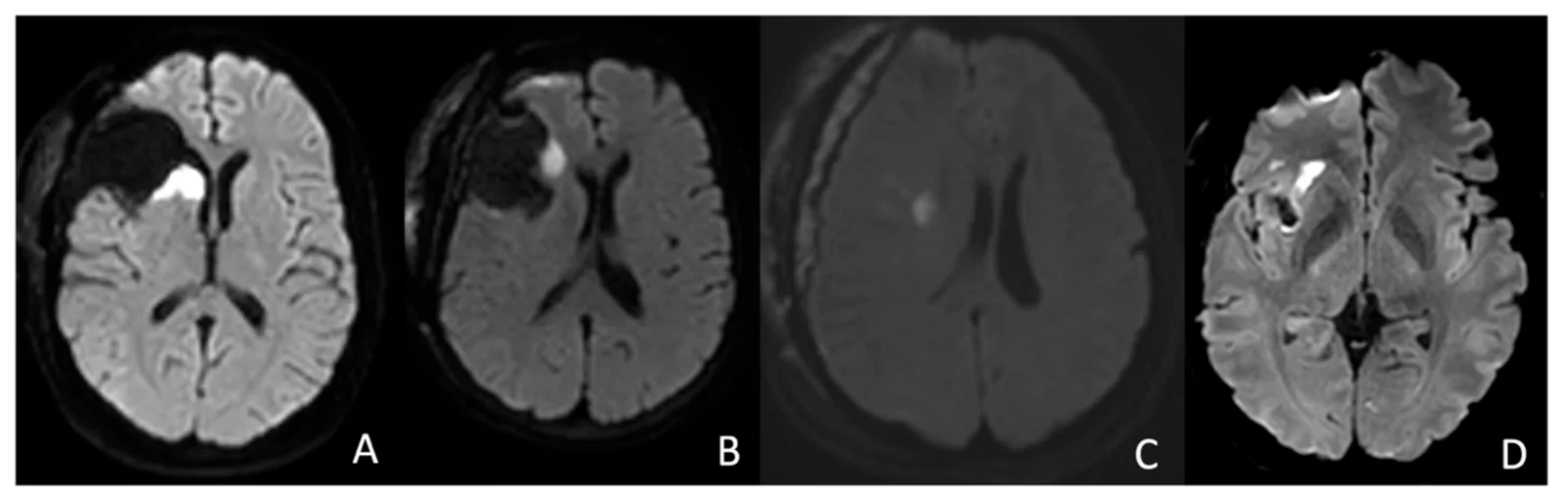
**Postoperative ischemic lesions in patients without motor deficits.** (**A**) Ischemic lesion in the territory of the recurrent artery of Heubner. (**B**) Ischemic lesion involving the anterior corona radiata, consistent with involvement of a LPA-MCA. (**C**) Ischemic lesion in the middle portion of the corona radiata, also corresponding to an LPA-MCA territory, without an apparent involvement of the CST based on its anatomical location. (**D**) Additional example of ischemia in the anterior corona radiata without associated motor deficit. **Abbreviations:** LPA, long perforating artery; MCA, middle cerebral artery; CST, corticospinal tract.

**Figure 3:**
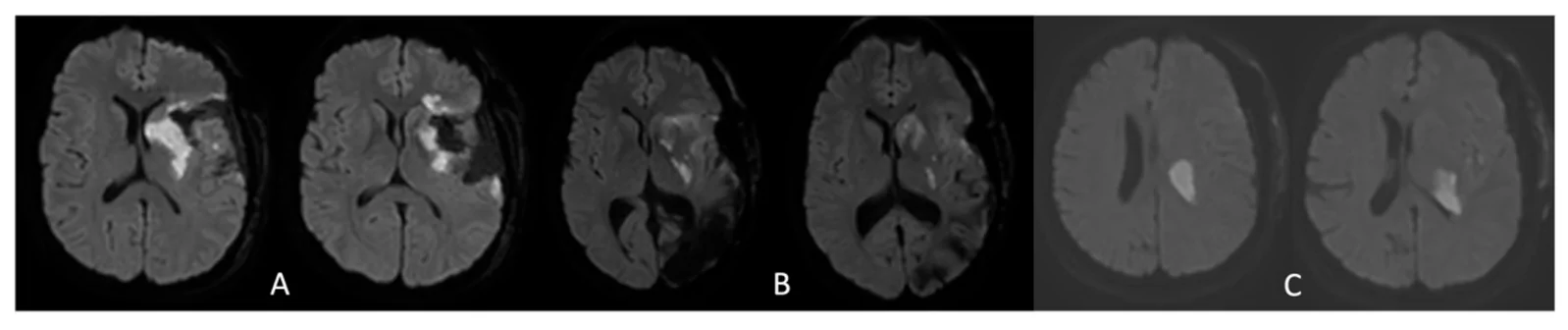
**Immediate postoperative DWI showing ischemic lesions associated with persistent neurological deficits at 3 months.** (**A**) Ischemic lesion involving the basal ganglia and posterior limb of the internal capsule (IC), consistent with injury to the LLA. (**B**) Additional example of ischemia in the territory of the LLA. (**C**) Ischemic lesion in the corona radiata, consistent with involvement of LPA-MCA. **Abbreviations:** DWI, diffusion-weighted imaging; IC, internal capsule; LLA, lateral lenticulostriate arteries; LPA, long perforating artery; MCA, middle cerebral artery.

Several observations support a potential vascular contribution to postoperative motor deterioration. First, some ischemic lesions were located away from the resection cavity (Figure 2 and Figure 3), suggesting that direct mechanical injury at the surgical margin alone may not fully account for the postoperative deficits. Second, intraoperative MEP deterioration or loss was observed in some cases during resection, without necessarily coinciding with direct subcortical manipulation of the motor pathway. Third, in three patients, resection proceeded to subcortical stimulation thresholds of 3 mA, indicating close proximity to the CST and therefore leaving open the possibility of concomitant direct injury. Conversely, postoperative ischemic lesions and persistent motor deficits also occurred in patients with subcortical stimulation thresholds above 5 mA. Furthermore, although tumors involving Berger–Sanai zones II and/or III were associated with lower subcortical stimulation thresholds, this anatomical distribution was not associated with a higher incidence of persistent motor deficits. Taken together, these observations suggest that proximity to the CST alone may not explain postoperative motor deterioration and support a potential contribution of vascular injury.

Our findings suggest that vascular preservation represents an important additional consideration, beyond functional mapping, when pursuing maximal safe resection in the insular region.

The vascular anatomy of the insular region is complex and highly relevant in the context of glioma surgery. The arterial supply of the insula is primarily derived from short perforating arteries arising from the M2 and M3 segments of the middle cerebral artery (MCA), whereas long perforating arteries, representing only 3–5% of MCA perforators, do not supply the insula itself but instead traverse it to irrigate the corona radiata [36]. Microanatomical studies have reported a median of approximately 4–5 long perforating arteries per hemisphere [37]. In the intraoperative setting, these vessels can be difficult to distinguish from short perforators, although they are typically characterized by a larger caliber and a non-tapering course [37,38].

Despite their relatively low number, long perforating arteries have considerable surgical relevance. Injury to these vessels—particularly in the posterior and superior aspects of the insula—may result in ischemic lesions involving the corticospinal tract (CST) within the corona radiata, leading to motor deficits [36,37]. Conversely, infarctions that spare the CST may occur without clinically evident motor impairment, reflecting the variability of their functional impact.

Another critical vascular structure is the group of the LLA, which enter the anterior perforated substance through its lateral aspect and supply the basal ganglia and internal capsule [39,40]. Their injury is a well-recognized cause of postoperative motor deficits due to their direct contribution to the vascularization of the posterior limb of the internal capsule, where the corticospinal tract is densely concentrated. In contrast, perforating branches arising from the anterior cerebral artery, including the recurrent artery of Heubner, enter the anterior perforated substance medially [40,41] and predominantly supply the head of the caudate nucleus, anterior putamen, and anterior limb of the internal capsule [40]; their injury is more commonly associated with cognitive or behavioral disturbances than with significant motor deficits.

In our series, postoperative ischemic lesions were observed in 31% of patients; however, only 30% of these patients developed a motor deficit (Figure 2), highlighting the importance of the vascular territory involved. Infarcts affecting the LLA were associated with motor deficits and resulted in more severe functional impairment. However, the very small number of cases (*n* = 2) precludes reliable statistical inference or firm conclusions regarding the relationship between this vascular territory and motor outcome. In contrast, ischemic lesions involving the LPA-MCA were more frequent but less often symptomatic, with only 23.5% of patients developing motor deficits, which were generally less severe (Figure 3).

Although some studies have reported a higher risk of ischemia with the transsylvian approach [19] this association was not observed in our series, as also reported by other authors [10], except for tumors in zone II of the Berger-Sanai classification [10]. Likewise, no increased risk of ischemia was found in high-grade tumors in our series, despite their greater vascularity.

The incidence of postoperative ischemia in insular glioma surgery is well established, with reported rates ranging from 20% to 60% [10,19,21]. Preservation of perforating vessels is therefore of paramount importance, although it may be technically challenging, as perforating arteries are frequently encased or displaced by the tumor.

Microsurgical techniques such as early identification of the most lateral LLA to define the medial resection plane [38], dissection of the MCA prior to tumor resection, and subpial tumor removal have been described as strategies to reduce the risk of injury to perforating vessels [16]. Adjuncts such as intraoperative Doppler ultrasonography [42] or indocyanine green angiography [43] have been described by some authors as useful tools for their identification and preservation.

Our results have important surgical implications. While intraoperative mapping techniques allow reliable identification of functional boundaries and are essential to prevent direct injury to eloquent structures, they do not fully mitigate the risk of vascular complications. Overall, our findings indicate that persistent postoperative motor deficits were consistently associated with ischemic lesions, suggesting that vascular injury may play an important role in their development. Therefore, surgical strategies should emphasize meticulous dissection along the vascular planes of the MCA and its perforators, with particular caution in the posterior and superior portions of the insula, where these vessels are most frequently encountered.

Overall, our findings reinforce the concept that, when modern intraoperative mapping techniques are used, postoperative motor deficits after insular glioma surgery are more frequently related to vascular injury than to direct corticospinal tract damage. Preservation of deep perforating arteries should therefore be considered a critical determinant of functional outcome.

This study has several limitations. Its retrospective, single-center design and relatively small sample size limit the generalizability of the findings. In particular, the small number of postoperative motor deficits resulted in wide confidence intervals for the diagnostic performance of intraoperative MEP monitoring, limiting the precision of these estimates. Accordingly, the observed 100% positive predictive value should be interpreted cautiously and requires confirmation in larger cohorts.

Although the overall surgical strategy and IONM protocols remained broadly consistent throughout the study period, the potential influence of temporal variations in surgical experience and perioperative management cannot be completely excluded. Furthermore, some heterogeneity existed in the intraoperative functional assessment, as eight patients underwent fully awake surgery without continuous MEP monitoring. While this did not preclude their inclusion in the analysis of postoperative motor outcomes and ischemic lesions, it limited the uniformity of intraoperative neurophysiological assessment across the cohort. The observational nature of this study and the limited number of neurological events also preclude definitive causal conclusions regarding the relative contributions of vascular preservation and functional mapping to postoperative motor outcomes.

Another limitation is that the involvement of the CST by postoperative ischemic lesions was inferred from their anatomical distribution and their relationship with the known vascular territories of deep perforating arteries, rather than confirmed through systematic postoperative DTI tractography or spatial correlation with preoperative CST reconstructions. Preoperative DTI was used for surgical planning but postoperative DTI was not systematically acquired. Although DTI tractography itself has inherent technical limitations and does not provide a definitive anatomical representation of white matter pathways, direct involvement of the CST by ischemic lesions could not be systematically confirmed.

Finally, a detailed postoperative neuropsychological assessment was not systematically performed, limiting our ability to evaluate the impact of ischemic lesions on higher-order cognitive functions. Future studies incorporating comprehensive neuropsychological testing are warranted to better characterize these outcomes following insular glioma surgery.

## 5. Conclusions

In our series, postoperative motor deficits after insular glioma surgery were associated with postoperative ischemia, highlighting the importance of vascular integrity for postoperative motor function.

Intraoperative MEP monitoring provided real-time information on corticospinal tract function and showed high specificity and positive predictive value for postoperative motor deficits, although sensitivity was limited. Stable MEPs therefore did not completely exclude postoperative motor deficits, particularly in the presence of postoperative ischemia. Subcortical stimulation provided complementary information regarding the proximity of the corticospinal tract during resection.

The clinical impact of postoperative ischemia varied according to the vascular territory involved. LLA infarctions were associated with more severe motor deficits, whereas LPA-MCA infarctions were more frequent and generally associated with milder impairment.

These findings emphasize that maximal safe resection of insular gliomas requires integration of functional mapping with meticulous preservation of the deep perforating arteries. Vascular preservation should therefore be considered an essential component of the surgical strategy, particularly when tumors are located in close proximity to these vessels.

## Figures and Tables

**Table 1:** Baseline Patient and Tumor Characteristics.

Variable	Value
**Number of patients**	64
**Age at surgery, median (IQR), years**	47 (38–57.8)
**Sex**	
Male	38 (59%)
Female	26 (41%)
**Tumor hemisphere**	
Right	28 (43.8%)
Left	36 (56.2%)
**Preoperative tumor volume (cm^3^), median (IQR)**	58.2 (43.5–82.3)
**Histopathological diagnosis**	
Glioblastoma, IDHwt, CNS WHO grade 4	28 (43.75%)
Astrocytoma, IDHm, CNS WHO grade 3	13 (20.3%)
Astrocytoma, IDHm CNS WHO grade 2	11 (17.2%)
ODG IDHm and 1p-19q codel, CNS WHO grade 3	8 (12.5%)
ODG IDHm and 1p-19q codel, CNS WHO grade 2	2 (3.1%)
Astrocytoma, IDHm, CNS WHO grade 4	2 (3.1%)
**Tumor grade**	
High-grade gliomas	51 (79.7%)
Low-grade gliomas	13 (20.3%)

**Abbreviations:** IQR: interquartile range; wt: wild-type; m: mutant; CNS: central nervous system. WHO: World Health Organization; ODG: oligodendroglioma; codel: codeleted.

**Table 2:** Surgical Characteristics and Extent of Resection.

Variable	Value
**Surgical approach** ***n* (%)**	
Transopercular	44 (68.8%)
Transsylvian	20 (31.2%)
**Extent of resection**	
Median EOR % (IQR)	93.4 (87.4–100)
EOR ≥ 90% ***n*** (%)	39 (60.9%)
Gross total resection ***n*** (%)	22 (34.4%)
**Residual tumor volume**	
Median cm^3^ (IQR)	4.5 (0–8.1)
Residual < 5 cm^3^ ***n*** (%)	33 (51.6%)
Residual < 10 cm^3^ ***n*** (%)	51 (79.7%)

**Abbreviations:** EOR, extent of resection; *n*, number of patients; IQR, interquartile range.

**Table 3:** Distribution of Subcortical Motor Stimulation Thresholds.

Subcortical Stimulation Threshold	Patients (*n*)	Percentage (%)
Negative stimulation	8	14.3
>15 mA	2	3.6
10–15 mA	2	3.6
5–10 mA	19	33.9
≤5 mA	25	44.6
Total	56	100

**Table 4:** Relationship Between Intraoperative MEP Monitoring and Postoperative Motor Deficits.

MEP Status	Patients (*n*)	Motor Deficit at 3 Months	
		Yes	No
Stable MEP signals	53	2 (3.8%)	51 (96.2%)
MEP deterioration or loss	3	3 (100%)	0 (0%)
Awake surgery (no MEP)	8	1 (12.5%)	7 (87.5%)
Total	64	6 (9.4%)	58 (90.6%)

**Abbreviations:** MEP, motor-evoked potential.

**Table 5:** Postoperative Ischemia, Vascular Territory, and Motor Outcome.

Vascular Territory	*n*	Motor Deficit, *n* (%)	
		Yes	No
LPA-MCA	15	4 (26.7%)	11 (73.3%)
LPA-MCA + ACA	2	0 (0%)	2 (100%)
ACA (isolated)	1	0 (0%)	1 (100%)
LLA	2	2 (100%)	0 (0%)
**Total**	20	6 (30%)	14 (70%)

**Abbreviations:** LPA-MCA, long-perforating arteries of the middle cerebral artery; ACA, anterior cerebral artery; LLA, lateral lenticulostriate arteries;.

**Table 6:** Clinical, neurophysiological, and radiological characteristics of patients with persistent motor deficits at 3 months.

Patient	Age/ Gender	Hemisph	Berger–Sanai	cMEP	scMEP (mA)	Vascular Territory	Immediate Postop Motor Deficit	3 Months Motor Deficit		3 Months KPS
1	38 F	R	T	lost	3	LPA-MCA	hemiplegia	arm 4-/5 hand 4+/5	leg 4+/5 foot 4+/5	80
2	47 M	L	T	NP	NP	LLA	hemiplegia	arm 3/5 hand 0/5	leg 4-/5 foot 1/5	60
3	31 M	L	1+3+4	lost	3	LLA	hemiplegia	arm 4/5 hand 3/5	leg 4-/5 foot 0/5	60
4	55 M	L	1+4	normal	9	LPA-MCA	arm 0/5 leg 3/5	arm 3/5 hand 3/5	leg 4+/5 foot 4+/5	70
5	61 M	L	3+4	normal	12	LPA-MCA	arm 2/5 leg 2/5	arm 3/5 hand 3/5	leg 4+/5 foot 4+/5	70
6	48 M	L	T	lost	3	LPA-MCA	arm 0/5 leg 4-/5	arm 2/5 hand 1/5	leg 4+/5 foot 4+/5	60

Abbreviations: F, female; M, male; R, right; L, left; T, total insular involvement; LPA-MCA, long-perforating arteries of the middle cerebral artery; LLA, lateral lenticulostriate arteries; cMEP, cortical motor-evoked potential; scMEP, subcortical motor-evoked potential; KPS, Karnofsky Performance Status.; NP: Not performed; Hemisph: Hemisphere.

## Data Availability

The data presented in this study are not publicly available due to patient privacy and confidentiality restrictions. Access to the de-identified data may be considered upon reasonable request to the corresponding author, subject to institutional ethical approval and applicable data protection regulations.
